# Validity and reliability of fluoroscopy for digital radiography: a new way to evaluate diaphragmatic mobility

**DOI:** 10.1186/s12890-017-0402-x

**Published:** 2017-04-17

**Authors:** Bruna Estima Leal, Márcia Aparecida Gonçalves, Liseane Gonçalves Lisboa, Larissa Martins Schmitz Linné, Michelle Gonçalves de Souza Tavares, Wellington Pereira Yamaguti, Elaine Paulin

**Affiliations:** 1 Physical Therapy Department,Santa Catarina State University (UDESC), Florianopolis, SC Brazil; 2 Lamina Medicine Diagnostis Clinic, Florianopolis, SC Brazil; 3 Federal University of Santa Catarina, Florianopolis, SC Brazil; 4 Physical Therapy Department, Rehabilitation Service, Hospital Sírio Libanês, São Paulo, SP Brazil; 5 Santa Catarina State University (UDESC), Rua Pascoal Simone, 358, Coqueiros, Florianópolis, SC Brazil CEP: 88080-350

**Keywords:** Diaphragm, Fluoroscopy, Validity, Reproducibility of results

## Abstract

**Background:**

Fluoroscopy is considered the most accurate method to evaluate the diaphragm, yet most existing methods for measuring diaphragmatic mobility using fluoroscopy are complex. To assess the validity and reliability of a new evaluation method of diaphragmatic motion using fluoroscopy by digital radiography of healthy adults.

**Methods:**

Twenty-six adults were evaluated, according to the parameters: anthropometry and pulmonary function test. The evaluation of diaphragm mobility by means of fluoroscopy by digital radiography method was randomly conducted by two raters (A and B). The Pearson correlation coefficient and the intraclass correlation coefficient (ICC) were used to assess the concurrent validity. The inter-rater and intra-rater reliability of the measurement of diaphragmatic motion was determined using ICC and a confidence interval of 95%.

**Results:**

There was a relationship in the assessment of the concurrent validity. There was good inter-rater reliability for right hemidiaphragm mobility and moderate reliability for left hemidiaphragm in the first assessment. In the second assessment, there was good reliability for the mobility of both hemidiaphragms. There was good intra-rater reliability in the mobility of both hemidiaphragms for raters A and B.

**Conclusion:**

The evaluation of diaphragmatic motion using fluoroscopy by digital radiography proved to be a valid and reliable method of healthy adults.

## Background

Specifically evaluating the mobility of the diaphragm is important for understanding and diagnosing possible alterations in the muscle, which can be compromised in several ways: due to central or peripheral nervous system dysfunction, muscular disease and thoracic or abdominal disease, resulting in a reduction of mobility or paralysis [1–6].

In clinical practice it is essential to use valid and reliable methods for assessing diaphragmatic dysfunction, because the use of subjective methods may compromise the results. Therefore, it is extremely important that any assessment method have its validity and reliability tested to ensure that the error in the measurement is reduced [7–9].

There are several imaging methods that assess diaphragmatic mobility: fluoroscopy [10–13], ultrasound [14, 15], computed tomography [16], magnetic resonance [17–19], and chest radiography [20, 21]. Each technique has its particularities in the observation of the diaphragm, considering cost, radiation exposure and method availability in the study environment [5, 22].

Considering all the methods for evaluating diaphragmatic mobility, the fluoroscopy is the most accurade method for assessing the diaphragm muscle because it provides dynamic images of the diaphragm and direct visualization of diaphragmatic movements in real time [5]. However, there are no studies confirming the validity and reliability of digital radiography fluoroscopy to assess diaphragmatic excursion.

Diaphragmatic mobility can be measured by the fluoroscopy method in various ways, but those forms reported in the literature are not simple to be obtained [10–13]. Some measures require radiographic impression for analysis [13], others, those recorded on video [10, 11], are not always available on fluoroscopy devices, and in other measurements, image calculations involve several complex lines for obtaining the value of diaphragmatic mobility [13].

Since fluoroscopy assesses diaphragm motion in real time [5], and the ways reported in the literature for measuring diaphragm mobility by means of fluoroscopy are complex [10–13], and there is no literature studies investigating its validity and reliability, we propose creating a new method and a new measurement procedure that is much more easily obtained in clinical practice, using fluoroscopy by digital radiography.

Thus, the aim of this study was to assess the validity and reliability (inter- and intra-rate) of a new method of evaluation of diaphragmatic mobility using fluoroscopy by digital radiography.

## Methods

### Sample

In this study, 26 apparently healthy adults were included in a convenience sample. They were recruited among students and employees of the Universidade do Estado de Santa Catarina (Brazil), as well as their relatives. The sample size calculation was based on Bonett [23] and Fleiss [24] studies. According to these authors, to assess test reliability, a sample can vary between 15 and 20 participants.

Inclusion criteria for the study were: non-smoking healthy subjects with normal pulmonary function (FVC and FEV_1_ ≥ 80% predicted and FEV_1_/FVC ≥ 0.7) without cardiorespiratory or neurological diseases, women who were not pregnant or with suspected pregnancy, participants without a diagnosis of cancer, or disease history or any other alteration that could impair the evaluations. Exclusion criteria were: participants who presented clinical complications of respiratory nature, inability to perform any of the procedures used in the study (lack of understanding or collaboration), clinical complications of the respiratory system and those who requested exclusion from the study. This study was approved (16696413.8.0000.0118) by the Research Ethics Committee of the Universidade do Estado de Santa Catarina (UDESC), Brazil, and all participants provided written informed consent.

### Study design

This is a cross-sectional study, with validity and reliability test, which assessed the agreement degree of diaphragmatic mobility by means of X-ray digital fluoroscopy [25].

Initially, anthropometric and pulmonary parameters were evaluated. Following, digital X-rays were scheduled in order to measure diaphragmatic excursion. Before the fluoroscopy examination, training of the diaphragm through diaphragmatic breathing exercise was performed, slow vital capacity (SVC) was also measured before and during the examination.

The examination of diaphragmatic motion by digital radiography fluoroscopy was randomly performed by two radiologists (raters A and B), and subsequently digital diaphragmatic mobility (DM_dig_) was measured by both raters, aiming to evaluate the intra-rater and inter-rater reliability. Diaphragmatic mobility by distance (DM_dist_) [20] was measured by rater A, to evaluate the validity of the method.

### Collection procedures

#### Anthropometry

For measurement of body mass and height, a previously calibrated scale and a stadiometer (*Welmy*® model W200/5) were used respectively. Once the anthropometric values were obtained (weight and height), the body mass index (BMI) was calculated using the equation: *body mass/(height)* ^*2*^ (kg/m^2^). Subjects were classified according to BMI as underweight (≤18.5 kg/m^2^), normal (18.5–24.9 kg/m^2^), overweight (25–29.9 kg/m^2^) and obese (≥ 30 kg/m^2^) [26].

#### Pulmonary function test

The pulmonary function test was performed using a previously calibrated spirometer in accordance with the methods and criteria recommended by the American Thoracic Society [27]. For assessment of forced vital capacity (FVC), forced expiratory volume in the first second (FEV_1_), and the FEV_1_/FVC ratio, a portable digital EasyOne® spirometer of the *ndd* brand was used. The criteria for normal lung test consisted in FVC and FEV_1_ ≥ 80% predicted and FEV_1_/FVC ≥ 0.7. Spirometric variables were expressed as absolute values and as percentages of predicted normal values, according to Pereira et al. [28].

Before performing the pulmonary function test, pulse oxygen saturation (SpO_2_) and heart rate (HR) were measured with the participant in the supine position and at rest with a pulse oximeter (Oximeter, Model MD300C11).

#### Assessment of diaphragmatic mobility by digital radiography fluoroscopy

Diaphragmatic mobility was assessed through examination of digital X-ray fluoroscopy in anteroposterior incidence (AP) by two raters. To perform the test, a Siemens fluoroscopy device, model Lumino RF Classic was used at a distance of 1.15 m from the image intensifier and X-ray tube.

Subjects were placed on a radioscopic table in a supine position with their feet supported to restrict their movement on the table, and a radiopaque graduation ruler was placed under the subjects’ trunks in a longitudinal direction and in the craniocaudal direction. Before performing the fluoroscopy by digital radiography to evaluate the diaphragmatic motion, training in diaphragmatic breathing was provided. The goal was to develop diaphragmatic proprioception movement and enable the evaluation of diaphragm maximum amplitude during fluoroscopic digital X-ray examination. Afterwards, we measured slow vital capacity (SVC) using a Wright Respirometer Brit.® UK ventilometer, before and during the examination of digital X-ray fluoroscopy with subjects in a supine position. Three manoeuvers were performed before radiographic exposure, and the highest value was recorded for later comparison with what would be assessed during the examination of diaphragmatic mobility. During the examination of digital X-ray fluoroscopy, each rater asked the subjects, while exhaling, to breathe using TLC (total lung capacity) until they approached RV (residual volume), and then, upon exhaling, to breathe from RV until approaching TLC. The values of the SVC manoeuvers obtained were compared to each other (before and during the exam), to determine whether the subjects performed the same respiratory effort before and during the evaluation of diaphragmatic mobility. If there was a difference greater than 10% from the previously obtained value, the examination would be repeated on the same day.

Subjects were evaluated randomly, in a simple raffle, by two expert radiologists (raters A and B) who guided subjects in a standardized way. The raters viewed the diaphragm movement through the display on the fluoroscopy device, which was positioned on the central part of the thorax, viewing both hemidiaphragms at the same time. The images of maximum expiration and inspiration were recorded on the same film, which remained motionless during exams.

#### Digital measurement of diaphragmatic mobility (DMdig)

**Fig. 1:**
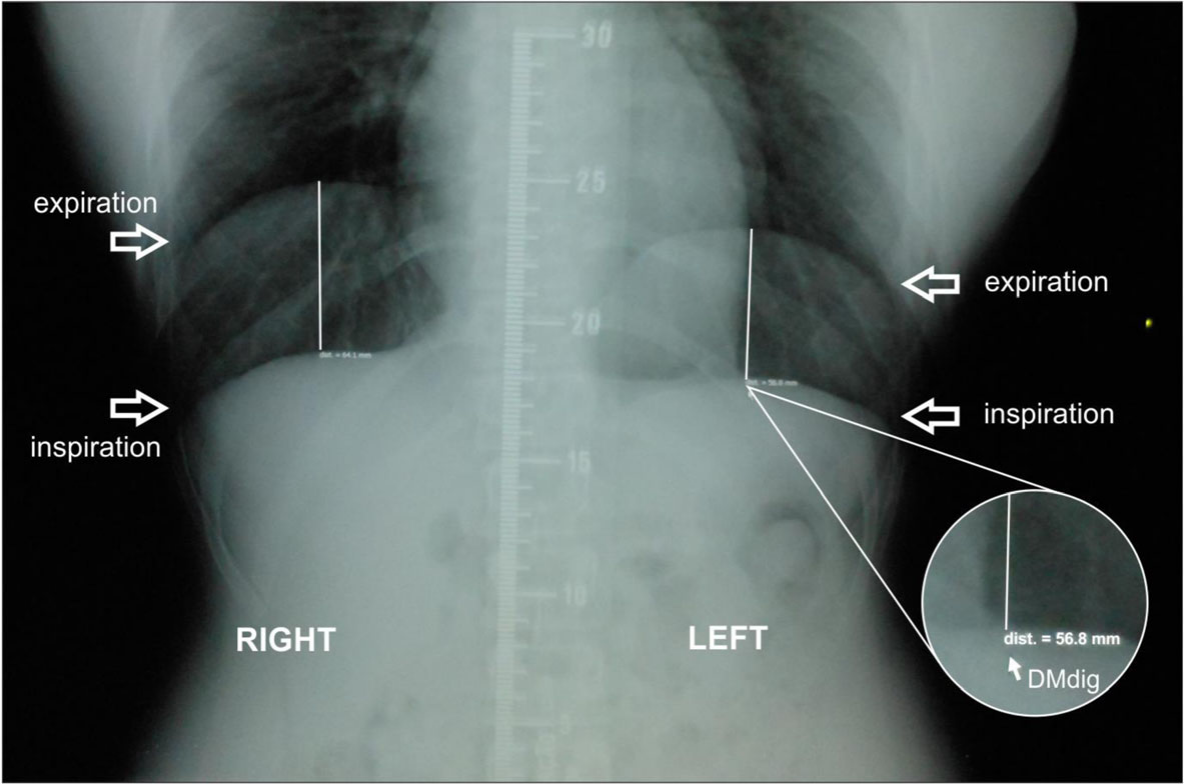
Measurement of diaphragm mobility obtained by the software. Digital radiography of the chest in anteroposterior view (AP) during maximal expiration and maximal inspiration conducted on the same film. Measurements of the mobility of *right* and *left* hemidiaphragms were obtained by the software of the device using the ruler on the image for calibration. Source: author's own production

Diaphragmatic mobility (DM_dig_) was measured by calculating the distance between the diaphragmatic dome in expiration and inspiration for the right and left hemidiaphragms, based on the method described by Saltiel et al*.* [20]. Initially, the line of MediWorks 8.4.215 software was calibrated using a radiopaque ruler to correct the magnification caused by the divergence of the rays. This calibration was performed by drawing a line with the computer cursor over a distance of 10 mm on the image of the ruler in the digital radiography, thus determining the actual distance. Then, to measure diaphragmatic mobility, the highest point on the hemidiaphragm in expiration was found and a straight perpendicular line was drawn with the cursor until it met the hemidiaphragm in inspiration finding the distance between the diaphragmatic domes (Fig. 1). This measurement was performed for both right and left hemidiaphragms (RH) and (LH).

#### Measurement of diaphragmatic mobility by distance (DM_dist_)

To measure DM_dist_, rater A identified, on the printed chest radiography, the highest point of the diaphragm during expiration of each hemidiaphragmatic dome, and from this point a line was drawn with a black marker until the hemidiaphragm during inspiration was found. The diaphragm mobility was determined by the distance between the diaphragm dome on expiration and inspiration by the calliper, both on the right side and the left side. To correct the image magnification caused by the divergence of X-rays, a correction formula was used: *Corrected mobility (mm) = mobility measure (mm) × 10/graduation on the ruler* (mm) [20].

#### Analysis of the validity

To assess the validity of the method (criterion validity), we analysed the concurrent validity. The competing method was the method of Saltiel et al. [20], which evaluates diaphragm mobility on the printed radiograph by distance (DM_dist_) by means of a calliper. Validity was assessed by relating the first measurement of DM_dig_ obtained by rater A with the measurement using the DM_dist_ method by the same rater. We evaluated both right and left hemidiaphragms.

#### Analysis of reliability

Measurements of diaphragmatic mobility were assessed soon after scanning the radiograph. The first measurement was used for the analysis of inter-rater reliability of the method (measurements A_1_ and B_1_) in the following manner: a measurement taken by rater A (A_1_) was correlated with that taken by rater B (B_1_). Then, a second measurement was performed for the analysis of inter-rater reliability of the measure as follows: rater A measured the digital radiography fluoroscopy exam performed by rater B, obtaining measure A_B_, and rater B measured the digital radiography fluoroscopy exam performed by rater A obtaining measure B_A_. The analysis was performed by correlating measures A_B_ with B_1_ and B_A_ with A_1_.

The intra-rater reliability of the measurement was assessed by measuring diaphragmatic mobility from the previous examination, after a minimum interval of 7 days and maximum of 20 days from the first measurement^25^. Both raters A and B measured the digital radiographs one more time (measures A_2_ and B_2_) performed at the beginning of the study. Inter-rater reliability was also examined in the second assessment.

Raters A and B did not know who conducted the radiographs and did not have access to the values of the other’s assessments. The results were analysed after completion of all assessments.

### Statistical analysis

Data were analysed using SPSS for Windows, version 20.0 (IBM SPSS Statistics, IBM, Armonk, NY, USA) and GraphPad Prism 5.1 program and treated with descriptive analysis (mean and standard deviation) and inferential analysis. Shapiro-Wilk test was used to analyse data normality.

Pearson correlation coefficient and intraclass correlation coefficient (two-way random model, with absolute agreement - ICC_[2.1]_) were used to assess the correlation between the digital method (DM_dig_) and the method of distance (DM_dist_.). The analyses of inter-rater and intra-rater reproducibility were determined using intraclass correlation coefficient (two-way random model, with absolute agreement - ICC_[2.1]_) and confidence interval (CI) of 95% [8]. Reliability was interpreted as the magnitude of Portney and Watkins coefficient of reliability [8]: ‘poor’ for coefficients under 0.50, ‘moderate’ for coefficients between 0.50 and 0.75, and ‘good’ for coefficients above 0.75. ICC varies from 0.00 to 1.00, and values close to 1.00 show strong reliability. Bland-Altman plot was also used to allow better visualization of agreement between the individual measures [29].

Wilcoxon test was used to compare the values of slow vital capacity before and during radiographic exposures for each rater. Paired T test was used to compare the mobility of the right and left hemidiaphragms. The T test for independent samples was used to compare the values of diaphragmatic mobility between male and female participants. The significance level for statistical treatment was 5% (*p* < 0.05).

## Results

Twenty-six healthy adults, 17 women (65.4%) and 9 men (34.6%) were evaluated, with a mean age of 28.19 ± 6.1 years, mean BMI 23.89 ± 4.2, classified as normal, healthy and with normal pulmonary function (Table 1). No subject refused to participate or desisted during assessments.

**Table 1:** Anthropometric and cardiopulmonary characteristics of the study participants

Variables	Average ± standard deviation variables (*n* = 26)
Age (years)	28.19 ± 6.1
Body mass (kg)	68.14 ± 16.7
Height (m)	1.68 ± 0.1
BMI (kg.m^- 2^)	23.89 ± 4.2
HR (bpm)	72.61 ± 9.7
SpO_2_ (%)	98.35 ± 0.6
FVC (% predicted)	92.85 ± 7.7
FEV_1_ (% predicted)	94.73 ± 7.1
FEV_1_/FVC (L)	0.91 ± 0.2

Values were express as mean and standard deviation

*n* number of subjetcs, *kg* lbs, *m* meters, *BMI* body mass index, *HR* heart rate, bpm: beats per minute, *SpO2* oxygen saturation by pulse, *FVC* (%predicted): Estimated percentage of FVC, FEV1 (%predicted): Estimated percentage of forced expiratory volume in one second, *L* liters

**Fig. 2:**
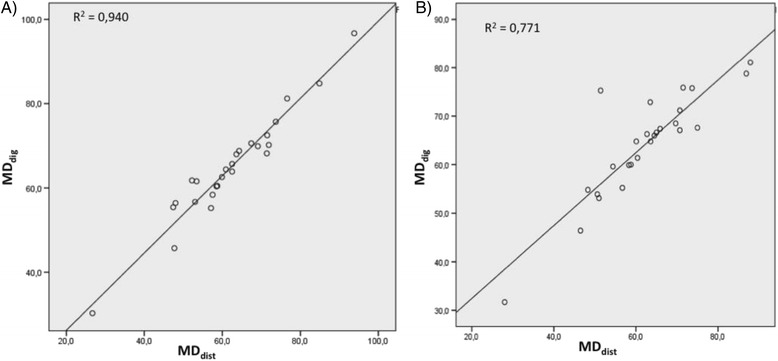
Correlation between DM_dig_ and DM_dist._ Correlation between methods DM_dig_ and DM_dist_ to assess the validity of the method (concurrent validity). The concurrent validity by relating the first measurement of DMdig obtained by rater A with the measurement using the DMdist method by the same rater. **a** Right hemidiaphragm. **b** Left hemidiaphragm. DM_dig_: digital diaphragmatic mobility. DM_dist_: diaphragmatic mobility by distance

A high correlation was found between DM_dig_ and DM_dist_ for the mobility of the right hemidiaphragm (RH) (*r* = 0.97, *p* = 0.00) and the left one (LH) (*r* = 0.88, *p* = 0. 00). There was good reliability for mobility in both hemidiaphragms (RH: ICC_[1, 2]_ = 0.98, 95% CI = 0.96 to 0.99; LH: ICC_[2,1]_ = 0.93, 95% CI = 0.84 to 0.97) (Fig. 2).

In the inter-raters analysis, in the first assessment there was good reliability for RH and moderate in LH. In the second assessment, there was good reliability for mobility in both hemidiaphragms. There was good intra-rater reliability of mobility in both RH and LH for rater A. Similar results were found in the measurements obtained by rater B in assessing RH and LH. The analyses of inter-rater and intra-rater reliability for digital diaphragmatic mobility of RH and LH are described in Table 2.

**Table 2:** Inter-rater and intra-rater reliability of the mobility measurement of the right and left hemidiaphragms method

Variables		ICC[2,1]	CI 95%
Right hemidiaphragm			
Inter-rater reliability	1ª assess	0.89	0.76–0.95
	2ª assess	0.84	0.68–0.93
Intra-rater reliability	rater A	0.83	0.66–0.92
	rater B	0.89	0.76–0.95
Left hemidiaphragm			
Inter-rater reliability	1ª assess	0.73	0.48–0.87
	2ª assess	0.78	0.56–0.89
Intra-rater reliability	rater A	0.86	0.70–0.93
	rater B	0.83	0.65–0.92

*ICC* [2,1] the intraclass correlation coefficient (two-way random model, with absolute and agreement), *CI 95%* confidence interval of 95%

To demonstrate higher reliability, we also evaluated inter-rater reproducibility of the measurement, where rater A measured the examination performed by rater B, yielding measure A_B_ and we compared this with measure B_1_. For this analysis there was good reliability for the ratings of RH and LH (ICC_[2,1]_ = 0.98, 95% CI = 0.96 to 0.99; ICC_[2,1]_ = 0.95, 95% CI = 0.90 to 0.98, respectively). Rater B also measured the examination performed by rater A to obtain measure B_A_. When comparing it with measure A_1_, there was good reliability for RH (ICC_[2,1]_ = 0.98, 95% CI = 0.96 to 0.99) and for LH (ICC_[2,1]_ = 0.86, 95% CI = 0.72 to 0.94).

**Fig. 3:**
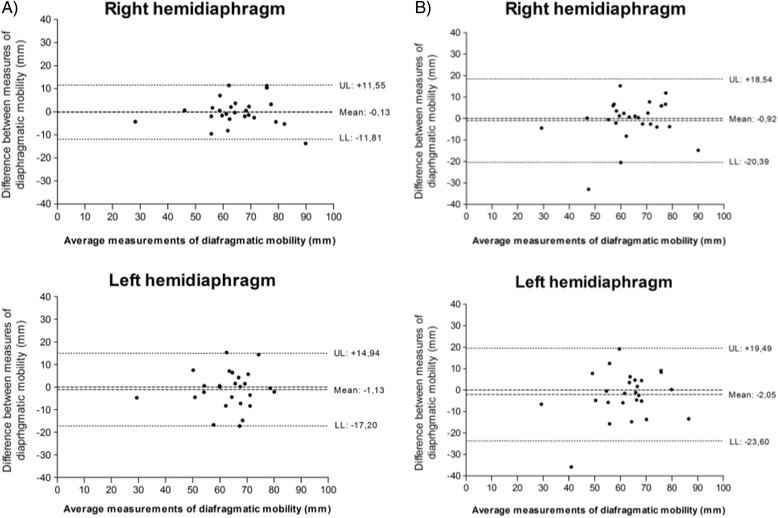
Inter-rater agreement between mobility measures (raters A and B) in 1^st^ and 2^nd^ assessments. Bland-Altman plot for the agreement between mobility measures of *right* and *left* hemidiaphragms, obtained by raters A and B (inter-rater agreement). **a** Interraters analysis 1^st^ assessment. **b** Interraters analysis 2^nd^ assessment. X axis: diaphragmatic mobility measurements mean obtained by raters in 1^st^ assessment ((*A*_*1*_ *+ B*_*1*_ *)/2*) and 2^nd^ assessment ((*A*_*2*_ *+ B*_*2*_ *)/2*). Y axis: difference between measures of diaphragmatic mobility, obtained by raters in 1^st^ assessment (*B*_*1*_ *- A*_*1*_) and 2^nd^ assessment (*B*_*2*_ *- A*_*2*_). SD: standard deviation UL: *upper limit* (*mean + 1.96 × SD*). LL: *lower limit* (*mean - 1.96 × SD*)

**Fig. 4:**
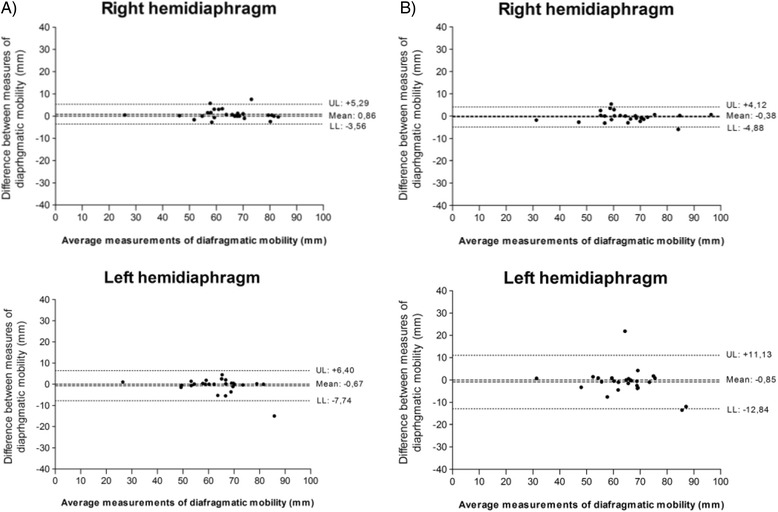
Intra-rater agreement between mobility measurements (rater A and B) in 1^st^ and 2^nd^ evaluations. Bland-Altman plot for the agreement between mobility measurements of right and left hemidiaphragms, obtained by rater A and rater B, in 1^st^ and 2^nd^ evaluations (intra-rater). **a** Rater A. X axis: Mean of diaphragmatic mobility measurements obtained by rater A, for each individual ((*A*_*1*_ *+ A*_*2*_ *)/2*). Y axis: difference between measures of diaphragmatic mobility, obtained by rater A, for each individual (*A*_*2*_ *- A*_*1*_). **b** Rater B. X axis: diaphragmatic mobility measurements mean obtained by rater B, for each individual ((*B*_*1*_ *+ B*_*2*_ *)/2*). Y axis: difference between measures of diaphragmatic motion, obtained by rater B, for each individual (*B*_*2*_ *- B*_*1*_). SD: standard deviation. UL: *upper limit* (*mean + 1.96 × SD*). LL: *lower limit* (*mean - 1.96 × SD*)

**Fig. 5:**
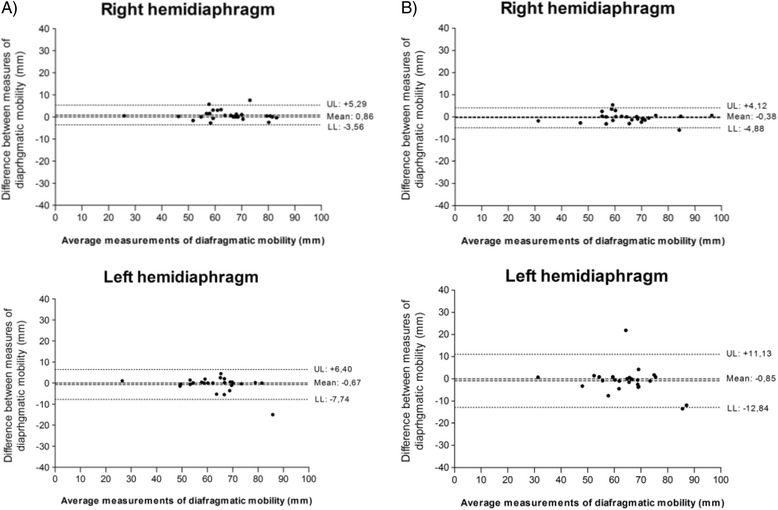
Inter-rater agreement of the measure between the mobility measurements obtained by raters **a** and **b**. Bland-Altman plot for the agreement between the mobility measurements of *right* and *left* hemidiaphragms, obtained by raters **a** and **b** (inter-rater agreement of the measures). X axis: diaphragmatic mobility measurements mean obtained by the raters **a** ((*A*_*B*_ *+ B*_*1*_ *)/2*) and **b** ((*B*_*A*_ *+ A*_*1*_ *)/2*). Y axis: difference between measures of diaphragmatic motion, obtained by raters **a** (*A*_*B*_ *- B*_*1*_) and **b** (*B*_*A*_ *- A*_*1*_). SD: standard deviation. UL: *upper limit* (*mean + 1.96 × SD*). LL: *lower limit* (*mean - 1.96 × SD*). A_B_: rater **a** measured the test conducted by the rater **b**. B_1_: rater **b** measured the 1^st^ test conducted by the rater **b**. B_A_: rater **b** measured the test conducted by the rater **a**. A_1_: rater **a** measured the 1^st^ test conducted by the rater **a**

According to Bland-Altman plot, there was good concordance between measures of mobility of both RH and LH, obtained by raters A and B (inter-rater agreement) (Fig. 3), and good concordance between measures of mobility of RH and LH, obtained by each of the raters (Fig. 4), at two different times (intra-rater agreement). Figure 5 shows that there was good concordance between measures of mobility of RH and LH, obtained by raters A and B when rater A measured the test conducted by rater B and when rater B measured the test conducted by rater A. There was good concordance because the difference between the measures is the limits of agreement (upper and lower limits). The mean of the measures between raters in all analyses is close to zero, which indicates reproducibility of the measurements. However, when analysing the inter-rater graphics, there was greater dispersion of the means obtained.

There were no differences between the manoeuvers of slow vital capacity (SVC) performed before and during fluoroscopy examinations by radiography by raters A and B, respectively (before: 3.92 ± 1.5 mm; during: 4.07 ± 1.5 mm, *p* = 0.57; before: 3.92 ± 1.5 mm; during : 4.12 ± 1.4 mm, *p* = 0.46).

The mobility measured in the right (RH) and left (LH) hemidiaphragms by the two raters (A and B) showed no statistically significant difference. The mean values of RH and LH mobility when analysed by rater A were 64.8 ± 12.6 mm (30.3 to 96.7 mm) and 64.1 ± 10.8 mm (31.7 to 92.3 mm), respectively and when analysed by rater B, 64.7 ± 12.5 mm (26.1 to 83.3 mm) and 62.9 ± 11.3 mm (27.0 to 81.5 mm), respectively. There was no statistically significant difference in the mobility of RH and LH measured by the two raters: A (*p* = 0.69) and B (*p* = 0.41).

## Discussion

This study demonstrated that the digital X-ray fluoroscopy method is valid and reliable for measuring mobility of the left and right hemidiaphragms in healthy adults. In clinical practice, the use of reliable instruments is essential for ensuring reliable results [9].

Fluoroscopy is the most accurate method for evaluating diaphragm dysfunction as it assesses the diaphragmatic motion in real time [5], but some measures require a video be made of the image [10, 11], which is not always available in fluoroscopy equipment, or they require calculations involving complex procedures to determine diaphragmatic motion [13].

Thus, the proposal of this study is innovative because it verifies the validity and the reliability of a new method of fluoroscopy and a new way of measuring diaphragmatic movement. For this, we applied a common resource used in clinical practice, which is the X-ray, and we innovated by using fluoroscopy associated with digital radiography. This method is easy to apply and measure, has a low cost because it is not necessary to print the radiography, and it can be another tool for professionals in the health assessment of patient’s diaphragmatic mobility.

Our proposal was to perform a digital measurement using software that is routinely used in medical radiological practice, but this is a unique way for evaluating diaphragmatic mobility in the scientific community. The measurement of diaphragmatic motion using the scanned image (DM_dig_) is simple and practical to perform, and proved to be reliable. We emphasize that the digitization of the exam is a technology that generates practicality, quality in analysis and makes storage easier in examinations, requiring no physical space. Moreover, it enables sharing among several professionals for the consultation and discussion of clinical cases.

This study found good reliability in the method analyses of diaphragmatic mobility for both raters A and B. There was good inter-rater reliability for the analysis of measure for RH and LH. Thus, we find that digital X-ray fluoroscopy is a reliable method for measuring diaphragmatic mobility, because a measure is considered reliable if the ICC is greater than 0.75 [8].

In the inter-rater analysis of the Bland-Altman plot in Fig. 3, a greater dispersion of the measures was observed. However, in Fig. 5 when rater A measured the test conducted by rater B and when rater B measured the test conducted by rater A, there was lower dispersion of data. Possibly, this difference in dispersion of data between the two analyses was due to the fact that, in the first measurement (Fig. 3), the rater performed the test and also assessed the measure.

In Fig. 5 the raters only had to perform the digital measurement of diaphragmatic mobility (DM_dig_) in the previously undertaken test. Therefore, we suggest that the same rater perform the measurement of diaphragmatic motion when comparing pre- and post-treatment, ensuring a lower bias between measurements, as our results show lower dispersion of data in intra-rater reliability.

When we observe the correlation between measures in the Bland-Altman plots (Figs. 3, 4 and 5), there was better agreement for the RH. This difference may have happened due to the proximity of the LH to the heart. Considering that the heart is a dynamic organ with involuntary contraction, at the time of the expiratory pause, a cardiac muscle contraction may have occurred during the evaluation by one of the raters, thereby altering the position of the left hemidiaphragm and consequently the location of the highest expiration point. However, it is important to note that the evaluation method of diaphragmatic mobility is reliable to assess both right and left hemidiaphragms, but the rater should choose the RH, if possible, because the agreement was better.

This study found a high variability in the amount of diaphragmatic motion (from 30.3 to 96.7 mm), similar to other studies [14, 15, 21]. Kantarci et al. [30] found variability of 25 to 84 mm for RH, and of 24 to 81 mm for LH, while in Gerscovich et al. [4], variability from 16.7 to 92 mm was found for RH, and 38 to 96 mm for LH. This variability may be related to BMI and age, because according to Kantarci et al. [30], subjects with low weight and younger than 30 have lower diaphragmatic mobility.

In this study there was no difference between diaphragmatic mobility for RH and LH, a result similar to data obtained in other studies by our group [20]. We also found no difference in diaphragmatic excursion between men and women. The studies by Bousseges et al*.* [15] and Kantarci et al*.* [30] found differences between diaphragmatic mobility between men and women. However, Grams et al. [14] and Saltiel et al*.* [20] found no such differences. This is possibly due to the sample size because the studies by Bousseges et al. [15] and Kantarci et al*.* [30] had a large number of participants (210 and 164 subjects, respectively), whereas Grams et al. [14] and Saltiel et al*.* [20] had a smaller number of about 40 subjects.

The mean values of RH mobility were 64.90 ± 12.9 mm, and 63.95 ± 11.7 mm for LH. According to Simon et al. [21], most healthy adults have diaphragm mobility ≥ 30 mm. Other studies have found amounts similar to those in our research of diaphragmatic mobility in healthy adults [12, 14]. There are, however, no reference values for diaphragmatic mobility in the literature.

When performing an examination, it is relevant to consider the acceptability and repeatability of the measurement. In the evaluation of diaphragmatic mobility by fluoroscopy, digital radiography is possible by analysing the acceptability of the examination to verify SVL; however, due to radiation it is not feasible to evaluate the repeatability of the measure. Despite this methodological limitation, it is possible to ensure the maximum movement of the diaphragm through the standardization of the evaluator’s guidelines, previous diaphragmatic training, and by obtaining the same SVL during the test.

In clinical practice, a change in diaphragm movement is often found, with a reduction in mobility or muscle paralysis, which may be due to several factors such as: changes in the muscle itself or neuromuscular conduction, dysfunction in the central nervous system, muscular dystrophy, muscle injury by trauma, phrenic nerve injury, chronic obstructive pulmonary disease, thoracic or abdominal diseases that interfere with their mobility, such as atelectasis or pleural effusions. The study of evaluation methods for diaphragmatic motion is important to better detect and diagnose diaphragm dysfunction [2–5]. A limitation of our study was to evaluate diaphragmatic mobility only in healthy subjects. Therefore, new studies for the validation of the method in patient populations would be relevant, aiming at creating a classification scale of diaphragm dysfunction severity.

Since the diaphragm is the most important respiratory muscle for pulmonary ventilation, it is essential to understand the functional condition of this muscle and its possible changes to establish therapeutic strategies to restore or improve its mobility and thus provide improved functionality and quality of life for the patient [1–3, 6].

## Conclusion

The results showed that fluoroscopy by digital radiography method is a valid and reliable instrument to assess the mobility of right and left hemidiaphragms of healthy adults.

## Abbreviations

1st: First evaluation
2nd: Second evaluation
AP: Anteroposterior incidence
BMI: Body mass index
Bpm: Beats per minute
CI: Confidence interval
DMdig: Diaphragmatic mobility digital
DMdist: Diaphragmatic mobility by distance
FEV1 (%predicted): Estimated percentage of forced expiratory volume in the first second
FEV1: Forced expiratory volume in the first second
FEV1/FVC: Ratio between forced expiratory volume in the first second and forced vital capacity
FVC (%predicted): Estimated percentage of forced vital capacity
FVC: Forced vital capacity
HR: Heart rate
kg: Kilograms
L: Litre
LH: Left hemidiaphragms
LL: Lower limit
M: Meter
mm: Millimeter
RH: Right hemidiaphragms
RV: Residual volume
SpO_2_: Oxygen saturation
SVC: Slow vital capacity
TLC: Total lung capacity
UL: Upper limit
ZCC: Intraclass correlation coeficiente

## References

[1] Yamaguti WP, Claudino RC, Neto AP, Chammas MC, Gomes AC, Salge JM (2012). Diaphragmatic breathing training program improves abdominal motion during natural breathing in patients with chronic obstructive pulmonary disease: a randomized controlled trial. Arch Physic Med Rehab.

[2] Kang HW, Kim TO, Lee BR, Yu JY, Chi SY, Ban HJ (2011). Influence od diaphragmatic mobility in hypercapnia in patients with chronic obstrutive pulmonar disease. J Korean Med Sci.

[3] Ayoub J, Cohendy R, Prioux J, Ahmaidi S, Bourgeois JM, Dauzat M (2001). Diaphragm moviment before and after cholecystectomy: a sonographic study. Anesth Analg.

[4] Gerscovich EO, Cronan M, McGahan JP, Jain K, Jones CD, McDonald C (2001). Ultrasonographic evaluation of diaphragmatic motion. J Ultrasound Med.

[5] Gierada DS, Slone RM, Fleishman MJ (1998). Imaging evaluation of the diaphragm. Chest SurgClin N Am.

[6] Reid WD, Dechman G (1995). Considerations when testing and training the respiratory muscles. PhysTher.

[7] Gadotti IC, Vieira ER, Magee DJ (2006). Importance and clarification of measurement properties in rehabilitation. Rev bras Fisioter.

[8] Portney GL, Watkins PM, Portney GL, Watkins PM (2008). Reliability. Foundations of clinical research application to pratice.

[9] Kimberlin CL, Winterstein AG (2008). Validity and reability of measurement instruments used in research. Am J Health Syst Pharm.

[10] Yi LC, Nascimento OA, Jardim JR (2011). Reliability of an analysis method for measuring diaphragm excursion by means of direct visualization with videofluoroscopy. ArchBronconeumol.

[11] Kleinman B, Frey K, VanDrunen M, Sheikh T, DiPinto D, Mason R, Smith T (2002). Motion of the diaphragm in patients with chronic obstructive pulmonary disease while spontaneously breathing versus during positive pressure breathing after anesthesia and neuromuscular blockade. Anestesiology.

[12] Unal O, Arslan H, Uzum K, Osbay B, Sakaria ME (2000). Evaluation of diaphragmatic movement with MR fluoroscopy in chronic obstructive pulmonary disease. J Clin Imaging.

[13] Verschakelen JA, Deschepper K, Jiang TX, Demedts M (1989). Diaphragmatic displacement measured by fluoroscopy and derived by respitrace. J Appl Physiol.

[14] Grams ST, Saltiel RV, Pedrini A, Mayer AF, Schivinski CIS, Paulin E (2014). Assesment of the reproducibility of the indirect ultrasound method of measuring diaphragm mobility. Clin Physiol Funct Imaging.

[15] Boussuges A, Gole Y, Blanc P (2009). Diaphragmatic motion studied by m-mode ultrasonography: methods, reproducibility, and normal values. Chest.

[16] Leung JC, Nance ML, Schwab CW, Miller WT (1999). Thickening of the diaphragm: a new computed tomography sign of diaphragm injury. J Thorac Imaging.

[17] Iwasawa T, Yoshiike Y, Saito K, Kagei S, Gotoh T, Matsubara S (2000). Paradoxical motion of the hemidiaphragm in patients with emphysema. J Thorac Imaging.

[18] Plathow C, Ley S, Fink C, Puderbach M, Heilmann M, Zuna I, Kauczor HU (2004). Evaluation of chest motion and volumetry during the breathing cycle by dynamic MRI in healthy subjects: comparison with pulmonary function tests. Investig Radiol.

[19] Kotani T, Minami S, Takahashi K, Isobe K, Nakata Y, Takaso M, Inoue M, Maruta T (2004). An analysis of chest wall and diaphragm motions in patients with idiopathic scoliosis using dynamic breathing MRI. Revista Spine.

[20] Saltiel RV, Grams ST, Pedrini A, Paulin E. High reliability of measure of diaphragmatic mobility by radiographic method in healthy individuals. Braz J PhysTher. 2013;17(2):128–36. doi: 10.1590/S1413-35552012005000076. 10.1590/S1413-35552012005000076 23778774

[21] Simon G, Bonnell J, Kazantzis G, Waller RE (1969). Some radiological observations on the range of movement of the diaphragm. Clin Radiol.

[22] Roberts HC (2009). Imaging the diaphragm. ThoracSurgClin.

[23] Bonett DG (2002). Sample size requirements for testing and estimating coefficient alpha. J Educ Behav Stat Winter.

[24] Fleiss JL (1999). Design and analysis of clinical experiments.

[25] Hulley SB (2008). Delineando a pesquisa clínica: uma abordagem epidemiológica.

[26] World Health Organization (2000). WHO obesity technical report series. obesity: preventing and managing the global epidemic. report of a world health organization consultation.

[27] Miller MR (2005). Series “ATS/ERS task force: standardisation of lung function testing”. Standardisations of spirometry. Eur Respir J.

[28] Pereira CAC, Rodrigues SC, Sato T (2007). Novos valores de referência para espirometria forçada em brasileiros adultos de raça branca. J Bras Pneumol.

[29] Bland JM, Altman DG (1986). Statistical methods for assessing agreement between two methods of clinical measurement. Lancet.

[30] Kantarci F, Mihmanli I, DemireL MK, Harmanci K, Akman C, Aydogan F (2004). Normal diaphragmatic motion and the effects of body composition: determination with m-mode sonography. J Ultrasound Med.

